# Navigating Vascular Anomalies in ALIF: Successful L3–L4 and L4–L5 Exposure in a Patient with Double Inferior Vena Cava

**DOI:** 10.1055/a-2964-9396

**Published:** 2026-11-11

**Authors:** Arthur Fiorotto de Mattos, Lucas Praxedes Chaves, Yunis Alexandre El Jaroush Palhavan, João Victor Martins Doro, Aécio Rubens Dias Pereira, Francisco Cialdine Frota Carneiro Júnior, Rafael Duarte de Souza Loduca, Paulo Mácio Porto de Melo

**Affiliations:** 1 Department of MedicalAnhembi Morumbi School of MedicineSão PauloSão PauloBrazil; 2 Department of NeurosurgeryHospital Militar de Área de São PauloSão PauloSão PauloBrazil; 3 Department of Medical 125102 Centro Universitário São CamiloSão PauloSão PauloBrazil; 4 Department of Vascular SurgeryHospital Militar de Área de São PauloSão PauloSão PauloBrazil

**Keywords:** duplicated inferior vena cava, vascular anomaly, anterior lumbar interbody fusion, intercaval corridor

## Abstract

Duplicated inferior vena cava (IVC) is a rare congenital vascular anomaly that may complicate retroperitoneal surgical approaches, particularly anterior lumbar interbody fusion (ALIF), due to altered venous anatomy and increased risk of vascular injury. A 30-year-old male with chronic refractory low back pain and multilevel disc degeneration underwent three-level ALIF. Preoperative imaging identified a duplicated IVC (type 2a). An extraperitoneal retroperitoneal approach was performed, allowing exposure of the L3–L4 and L4–L5 levels through the intercaval corridor. Although increased vascular complexity and enlarged venous tributaries were encountered, meticulous dissection and selective ligation enabled safe mobilization and adequate exposure. Estimated blood loss was 150 mL, operative time was 2 h and 50 min, and the postoperative course was uneventful, with discharge on postoperative day 3. Duplicated IVC does not represent a contraindication to ALIF, even in multilevel procedures. Careful preoperative identification, detailed anatomical understanding, and appropriate surgical technique are essential to minimize complications and achieve favorable outcomes.

## Introduction

Duplicated inferior vena cava (IVC) is a rare congenital vascular anomaly, most commonly identified incidentally on cross-sectional imaging, with a reported prevalence of up to 3% in the general population. 1 This condition results from the bilateral persistence of the supracardinal veins during embryologic development. 2 Although frequently asymptomatic, duplicated IVC carries important clinical implications, including an increased risk of deep venous thrombosis and pulmonary embolism. 3 Therefore, its recognition is essential for accurate radiologic interpretation and safe surgical planning.

Several anatomic subtypes of duplicated IVC have been described, based on the configuration and confluence patterns of the duplicated venous segments. 4 A thorough understanding of these variations is particularly important to minimize complications during retroperitoneal procedures, 5 especially those requiring anterior mobilization of major vascular structures, such as anterior lumbar interbody fusion (ALIF).

ALIF has become an established and effective technique for lumbar arthrodesis, particularly in cases where restoration of sagittal alignment is required. Compared with posterior approaches, including transforaminal lumbar interbody fusion (TLIF) and posterior lumbar interbody fusion (PLIF), the anterior approach offers wider exposure of the intervertebral disc space, enables more complete discectomy, and allows placement of larger interbody cages that facilitate restoration of disc height and lumbar lordosis. 6 However, in the presence of vascular anomalies such as duplicated IVC, anterior exposure poses additional challenges, requiring meticulous preoperative evaluation and, in some situations, specialized intraoperative strategies due to increased risk of iatrogenic injury and potentially complex vascular mobilization.

Accordingly, early identification of venous anomalies and the involvement of an experienced vascular surgeon are critical to ensuring procedural safety and optimizing surgical outcomes. 7

The aim of this report is to present a case of duplicated IVC identified during preoperative evaluation for ALIF, detailing the surgical strategy, intraoperative vascular management, and postoperative course at a specialized spine surgery center.

## Illustrative Case

A 30-year-old male patient, an active-duty military serviceman, presented with an 11-year history of chronic axial low back pain associated with radiation to the left lower limb and occasional paresthesia in a distribution consistent with the L5 dermatome.

He had previously undergone extensive conservative treatment, including analgesics, nonsteroidal anti-inflammatory drugs, and physical therapy, as well as diagnostic facet joint blocks followed by bilateral percutaneous facet rhizotomy from L3 to S1, without significant pain relief. The condition progressed to a refractory state, resulting in substantial limitation of activities of daily living, particularly those related to his military duties.

The patient denied any history of lumbar trauma, comorbidities, allergies, or substance use. His regular medications included acetaminophen 500 mg four times a day, pregabalin 150 mg twice daily, and tramadol 100 mg as needed.

Initial clinical evaluation revealed a patient in good general condition, alert and oriented to time and place, with isocoric and photoreactive pupils. No motor or sensory deficits were identified, and the Glasgow Coma Scale score was 15. Deep tendon reflexes were preserved and symmetrical. The straight leg raise test was positive on the left and negative on the right. Lumbar pain was elicited during trunk flexion, and palpation of the lumbar spine revealed localized tenderness predominantly at the L4 and L5 levels, with a reported pain intensity of 4 out of 10. The patient weighed 82 kg, was 1.84 m tall, and had a body mass index of 24.2 kg/m ^2^ .

Magnetic resonance imaging (MRI) of the lumbosacral spine demonstrated intervertebral disc degeneration, classified as Pfirrmann grade III at the L3–L4 level and Pfirrmann grade IV at the L4–L5 and L5–S1 levels ( Fig. 1 ). Given the refractoriness to conservative management, significant functional impairment, and multilevel degenerative involvement, a three-level ALIF with posterior percutaneous supplementation was indicated.

**Fig. 1:**
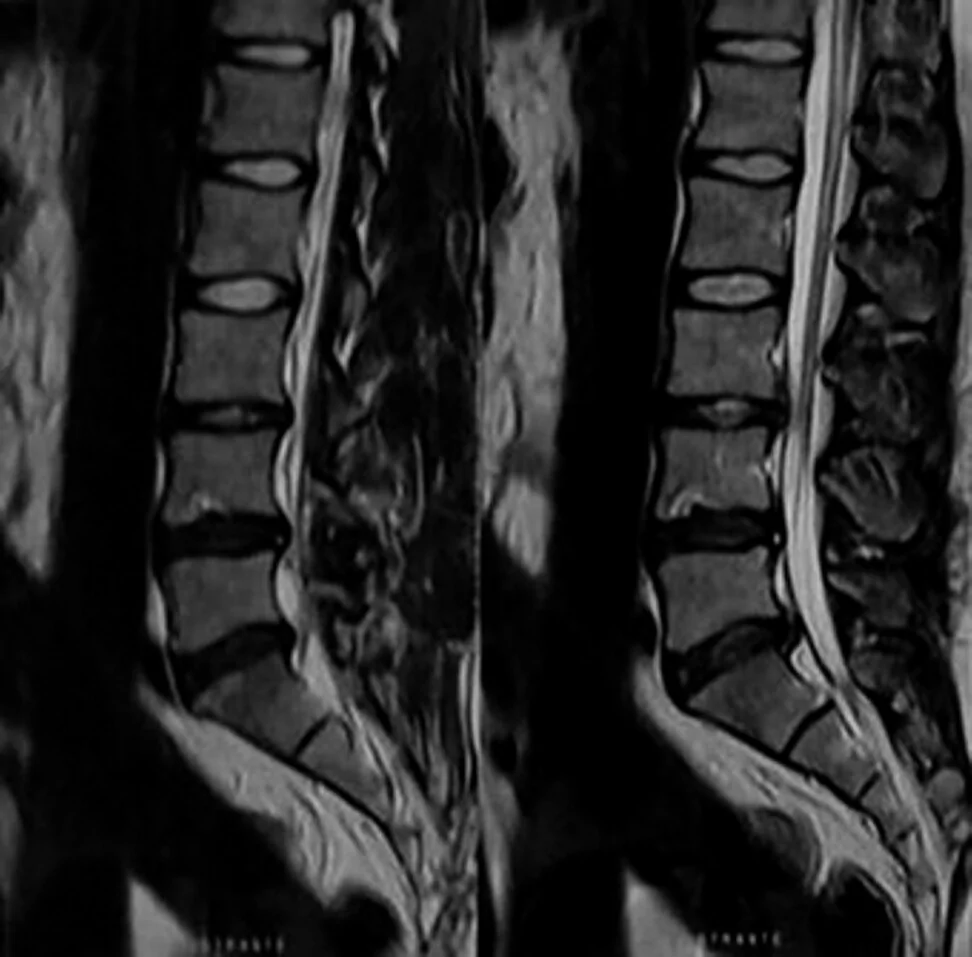
Sagittal T2-weighted magnetic resonance imaging of the lumbosacral spine demonstrating disc degeneration from L3 to S1.

The same MRI study revealed imaging findings suggestive of a duplicated IVC ( Fig. 2 ).

**Fig. 2:**
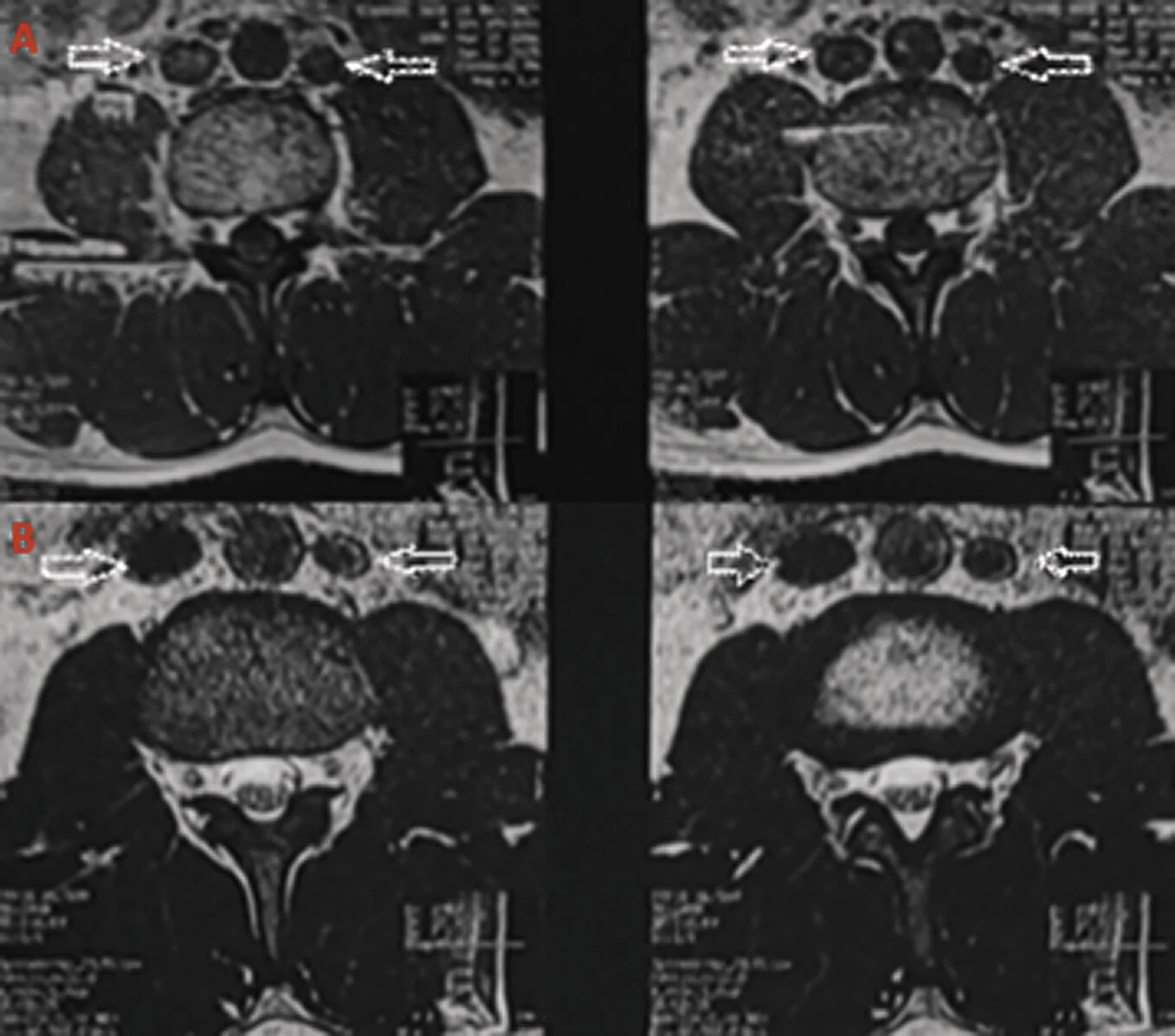
Axial T1-weighted ( **A** ) and T2-weighted ( **B** ) magnetic resonance imaging of the lumbosacral spine. Arrows indicate the right and left inferior venae cavae, with the abdominal aorta positioned between them.

A standard extraperitoneal retroperitoneal approach was performed to allow wide exposure of all three planned levels. Anterior access to the lumbar spine at the L3–L4 and L4–L5 levels was achieved through the interval between the two inferior venae cavae ( Fig. 3 ), requiring meticulous dissection due to increased venous fragility.

**Fig. 3:**
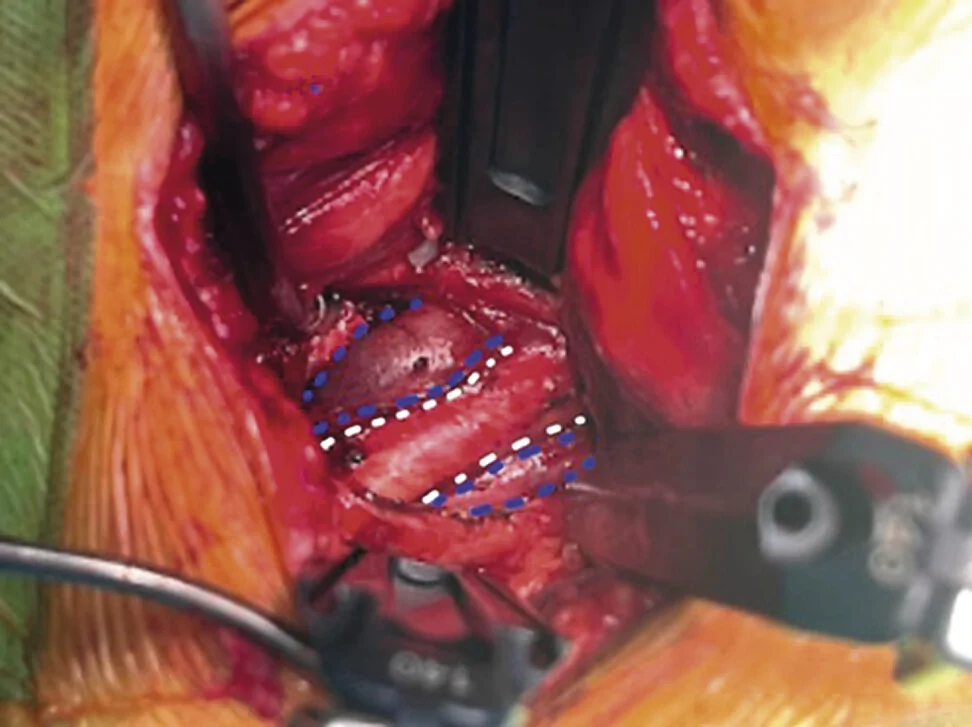
Intraoperative view demonstrating the intercaval corridor between the duplicated inferior venae cavae. The abdominal aorta is centrally positioned (white lines), with the right and left inferior venae cavae (blue lines) forming a reproducible surgical corridor for anterior lumbar exposure.

An increased number of venous tributaries and larger-caliber veins than typically encountered were observed, necessitating vessel ligation, as bipolar cautery alone proved insufficient to achieve adequate hemostasis. Intraoperative blood loss was estimated at approximately 150 mL, calculated using standard methods based on the volume collected in suction canisters and the weight of surgical sponges.

Additional abnormalities of the pelvic, retroperitoneal, and lumbar venous drainage were noted, with veins of greater caliber than usual. Despite these anatomical variations, the venous anomaly did not result in a significant increase in intraoperative blood loss and did not compromise adequate exposure of the intervertebral disc spaces. The total operative time was 2 h and 50 min.

On postoperative day 1, computed tomography (CT) of the lumbosacral spine was obtained ( Fig. 4 ). Bone window images ( Fig. 4A ) demonstrated appropriate positioning of the interbody cages, with an apparent gain in lumbar lordosis. Three-dimensional reconstruction ( Fig. 4B ) confirmed correct positioning of both the cages and pedicle screws, as well as satisfactory restoration of sagittal alignment.

**Fig. 4:**
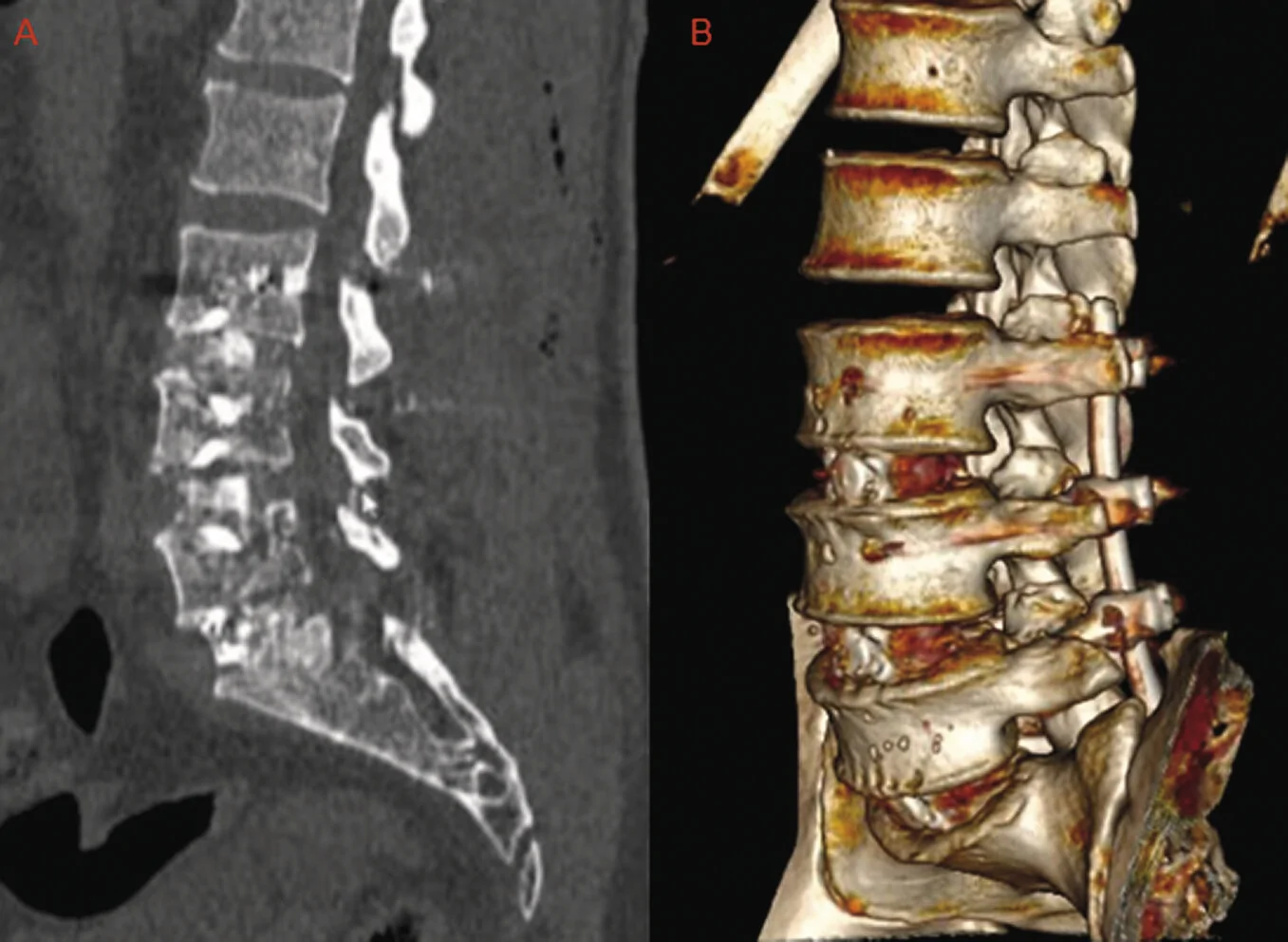
Postoperative computed tomography of the lumbosacral spine following three-level anterior lumbar interbody fusion (ALIF). ( **A** ) Sagittal bone window demonstrating proper positioning of the interbody cages and restoration of lumbar lordosis. ( **B** ) Three-dimensional reconstruction confirming appropriate placement of the interbody cages and percutaneous pedicle screws, with satisfactory sagittal alignment.

Coronal CT images further illustrated the spatial relationship between the interbody cages and the duplicated inferior venae cavae ( Fig. 5 ).

**Fig. 5:**
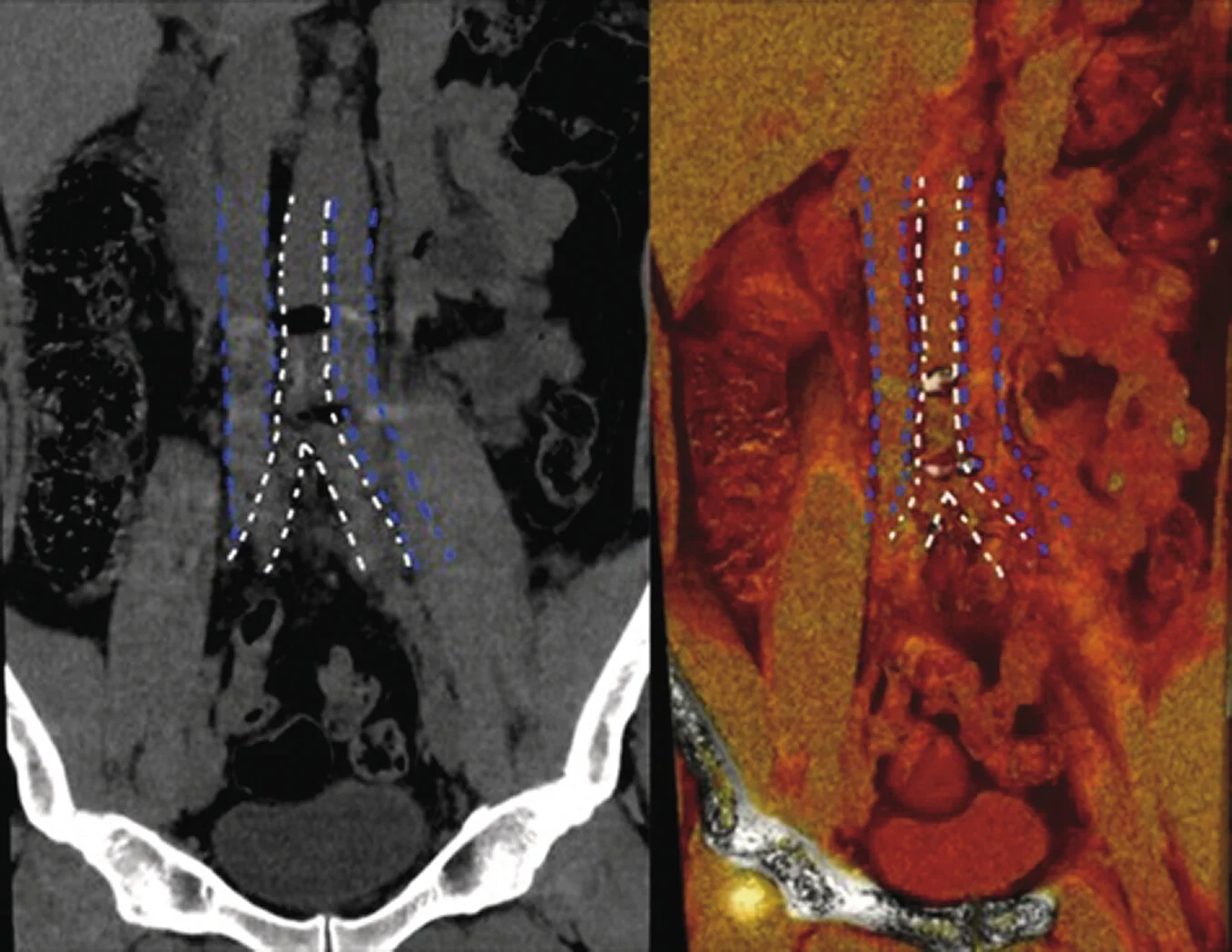
Immediate postoperative coronal computed tomography demonstrating the spatial relationship between the interbody cages and the duplicated inferior venae cavae. The cages at L3–L4, L4–L5, and L5–S1 are appropriately positioned, with the right and left inferior venae cavae (blue lines) coursing laterally to the implants and the abdominal aorta centrally located (white lines).

Postoperatively, an abdominal drain was maintained according to institutional protocol, with no evidence of excessive bleeding, and was removed on postoperative day 3. The patient had an uneventful recovery and was discharged on postoperative day 3 without the need for additional interventions.

## Discussion

Duplicated IVC, reported in up to 3% of the general population, results from the bilateral persistence of the supracardinal veins during embryologic development. 1 2 This vascular anomaly is most often asymptomatic and is frequently identified incidentally on cross-sectional imaging performed for unrelated reasons or during surgical procedures. In the present case, the patient was entirely asymptomatic with respect to the duplicated IVC, and the finding represented an incidental preoperative imaging diagnosis.

From a surgical standpoint, duplicated IVC may significantly complicate retroperitoneal approaches by altering normal venous anatomy, increasing the density and caliber of venous tributaries, and reducing the available avascular surgical corridors. 8 These factors collectively may elevate the risk of inadvertent vascular injury, uncontrolled venous bleeding, need for unplanned vascular repair, prolonged operative time, and, in severe cases, life-threatening hemorrhage. Additional challenges may include increased venous friability, difficulty achieving hemostasis with bipolar cautery alone, and limited options for safe vascular mobilization, particularly during multilevel anterior lumbar exposure.

Despite these theoretical risks, the patient described in this report did not experience excessive intraoperative blood loss or postoperative vascular complications. This favorable outcome underscores the importance of meticulous preoperative planning and careful intraoperative technique when dealing with complex venous anatomy.

Although duplicated IVC introduces anatomical complexity, the technical adjustments required in this case did not constitute a major surgical challenge. The primary modifications included: meticulous blunt and sharp dissection within the intercaval space to preserve venous integrity; early identification and ligation of enlarged venous tributaries, as bipolar cautery alone proved insufficient for reliable hemostasis; gentle and limited mobilization of the venae cavae, avoiding excessive traction to reduce the risk of venous tearing; close collaboration with an experienced vascular surgeon, ensuring immediate management capability in case of vascular injury.

These adaptations allowed safe anterior exposure of the L3–L4 and L4–L5 disc spaces between the two venae cavae. Importantly, exposure of the L5–S1 level, performed inferior to the vascular bifurcation, did not present additional difficulty, consistent with standard ALIF technique.

The concept of an intercaval surgical corridor, as illustrated in Figs. 3 and 5 , may represent a reproducible anatomical window for anterior lumbar exposure in select patients with duplicated IVC.

Collectively, these findings support the concept that duplicated IVC should not be considered a contraindication to ALIF, even in multilevel procedures, provided that adequate expertise and planning are in place.

According to the classification proposed by Morita et al 9 ( Fig. 6 ), several anatomic subtypes of duplicated IVC exist, based on the symmetry of the duplicated veins and their pattern of confluence. In type 2a duplication, the most common one, two symmetric infrarenal inferior venae cavae are present, typically draining independently into the renal veins, with a single suprarenal IVC continuing cranially. This configuration creates a relatively predictable intercaval space, which may be exploited surgically during anterior spinal exposure. Compared with other subtypes—such as asymmetric duplication or complex cross-venous communications—type 2a appears to be more favorable for anterior approaches, as it offers clearer anatomical planes and fewer aberrant venous crossings over the disc spaces.

**Fig. 6:**
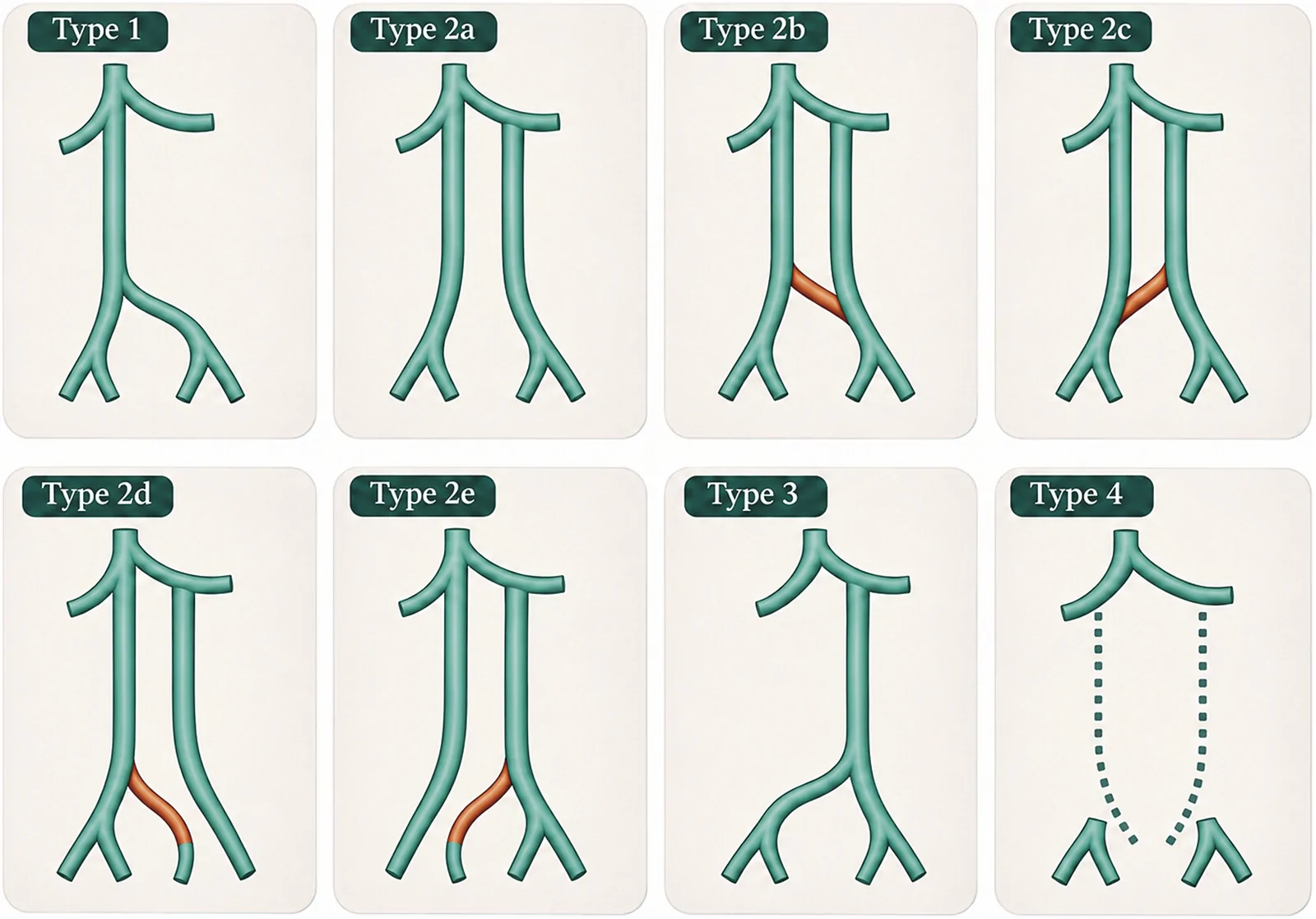
Classification of inferior vena cava duplication.

More complex subtypes, particularly those involving extensive venous interconnections, retroaortic renal veins, or anomalous iliac venous drainage, may pose higher surgical risk by further limiting mobilization and increasing the likelihood of vascular injury. In contrast, the type 2a configuration observed in our patient likely contributed to the relative technical feasibility of the procedure.

The clinical implications of duplicated IVC extend beyond intraoperative risk. Venous duplication may promote blood flow stasis, thereby predisposing patients to deep venous thrombosis, particularly in the postoperative period or during phases of reduced mobility. 10 Consequently, postoperative management should incorporate strategies specifically tailored to mitigate thromboembolic risk, including early mobilization as tolerated, appropriate pharmacologic thromboprophylaxis when not contraindicated, and the use of mechanical prophylactic measures such as intermittent pneumatic compression devices. A more aggressive thromboprophylaxis strategy may be justified in select cases.

In addition, heightened clinical vigilance for signs and symptoms of thromboembolic events—especially lower-limb edema or unexplained pain—is warranted.

Collectively, these considerations underscore the importance of recognizing venous anatomical anomalies as an integral component of individualized perioperative risk assessment and management.

To our knowledge, only one prior report has described the use of ALIF in a patient with duplicated IVC. Chapon et al reported a two-level ALIF (L4–L5 and L5–S1) performed via a right-sided retroperitoneal approach in a patient with chronic low back pain and S1 radiculopathy. 11 Similar to our case, the patient exhibited a type 2a duplicated IVC. The procedure was completed successfully without long-term complications, and the patient was discharged on postoperative day 3 with favorable clinical evolution. 11

When compared with the case by Chapon et al, our report expands the existing literature by demonstrating the feasibility of multilevel ALIF in the presence of duplicated IVC. Operative time in our report was 2 h and 50 min, compared with a mean of 2 h and 27 min reported among 47 patients undergoing two-level ALIF in a retrospective cohort study. 12 Postoperative recovery was comparable, further supporting the safety of the anterior approach in carefully selected patients with this vascular anomaly.

In contrast to the previously reported two-level ALIF, our case involved three-level exposure, which could inherently increase vascular manipulation and potential risk, further reinforcing the importance of detailed anatomical understanding.

A systematic review and meta-analysis evaluating the outcomes of ALIF for lumbar disc herniation reported a mean intraoperative blood loss of approximately 122 mL, reflecting the relatively low bleeding profile of this approach. 13 In our case, intraoperative blood loss was estimated at approximately 150 mL, calculated based on the volume collected in suction canisters and the weight of surgical sponges. This finding demonstrates a comparable bleeding profile, despite the presence of duplicated IVC and the need for multilevel exposure, further supporting the feasibility and safety of ALIF in such patients.

This study has inherent limitations. First, it represents a single case report, which precludes generalization of the findings or establishment of definitive clinical guidelines. Second, the absence of long-term follow-up limits conclusions regarding delayed vascular or thromboembolic complications. Additionally, the favorable outcome observed may be influenced by the specific anatomical subtype (type 2a), surgical expertise, and institutional experience, which may not be universally reproducible. An additional limitation of this report is that the ALIF was performed across three levels, a surgical extent that remains poorly represented in the current literature.

Therefore, while this report contributes valuable insight into the management of duplicated IVC during multilevel ALIF, its findings should be interpreted cautiously. Larger case series and comparative studies are necessary to better define risk stratification, technical recommendations, and long-term outcomes in this unique patient population.

## Conclusion

Recognition of anatomical variations of the IVC, such as duplication, is essential for safe surgical practice. Analysis of the present case demonstrates that although this anomaly is frequently asymptomatic, it may pose intraoperative challenges. Despite operative time and blood loss remaining within expected ranges and the procedure being considered safe, early recognition of this condition—combined with accurate interpretation of preoperative imaging—plays a decisive role in surgical planning and in reducing intra- and postoperative complications. Therefore, duplicated IVC does not appear to represent a prohibitive technical challenge for the anterior approach to the lumbosacral spine, including for three-level approaches, and should not be regarded as a contraindication when appropriate anatomical understanding and surgical expertise are present.

## Declaration of GenAI Use

No generative artificial intelligence tools were used in the preparation of this manuscript.
